# Polydeoxyribonucleotide in the Treatment of Tendon Disorders, from Basic Science to Clinical Practice: A Systematic Review

**DOI:** 10.3390/ijms24054582

**Published:** 2023-02-26

**Authors:** Davide Bizzoca, Giovanni Brunetti, Lorenzo Moretti, Andrea Piazzolla, Giovanni Vicenti, Francesco Luca Moretti, Giuseppe Solarino, Biagio Moretti

**Affiliations:** 1 UOSD Spine Surgery, AOU Consorziale Policlinico, 70124 Bari, Italy; 2 PhD. Course in Public Health, Clinical Medicine and Oncology, Department DiMePre-J, University of Bari “Aldo Moro”, Piazza Giulio Cesare 11, 70124 Bari, Italy; 3 Orthopaedics Unit, Department DiBraiN, School of Medicine, University of Bari “Aldo Moro”, AOU Consorziale Policlinico, 70124 Bari, Italy; 4 National Centre for Chemicals, Cosmetic Products and Consumer Protection, National Institute of Health, 00161 Rome, Italy

**Keywords:** PDRN, tendinopathies, tendon diseases, tendinitis, fasciitis, ESWT, musculoskeletal pain, lateral epicondylitis, rotator cuff disease, Achilles tendinopathy

## Abstract

Polydeoxyribonucleotide (PDRN) is a proprietary and registered drug with several beneficial effects, including tissue repairing, anti-ischemic action, and anti-inflammatory properties. The present study aims to summarize the current evidence about PRDN’s clinical effectiveness in the management of tendon disorders. From January 2015 to November 2022, OVID-MEDLINE^®^, EMBASE, Cochrane Library, SCOPUS, Web of Science, Google Scholar and PubMed were searched to identify relevant studies. The methodological quality of the studies was evaluated, and relevant data were extracted. Nine studies (two in vivo studies and seven clinical studies) were finally included in this systematic review. Overall, 169 patients (male: 103) were included in the present study. The effectiveness and safeness of PDRN has been investigated in the management of the following diseases: plantar fasciitis; epicondylitis; Achilles tendinopathy; pes anserine bursitis; chronic rotator cuff disease. No adverse effects have been recorded in the included studies and all the patients showed an improvement in clinical symptoms during the follow-up. PDRN are a valid emerging therapeutic drug in the treatment of tendinopathies. Further multicentric randomized clinical studies are needed to better define the therapeutic role of PDRN, especially in combined clinical protocols.

## 1. Introduction

Tendon injuries are major musculoskeletal disorders [1]. Tendinopathy is the preferred terminology for persistent tendon pain and loss of function related to mechanical loading as determined in a new consensus study [1].

The term “tendinopathy” characterizes a clinical condition underlying a picture of inflammation, pain, swelling and functional limitation of the tendon and the anatomical structures adjacent to it [1].

The incidence and prevalence of tendinopathy vary widely among the different body segments and according to age, sex, type of sports and physical activity, occupational setting and specific pathological condition.

Rotator cuff tendinopathy and tendinopathy of the elbow are the most common in the upper extremity [2,3]. On the other hand, the most frequent tendinopathies in the lower limb involve the heel (plantar fascia and Achilles tendon) and the knee (patellar tendon) [4].

The pathogenesis of tendinopathies is multifactorial and complex, and the exact cause is not always easy to be traced. Several studies have led to multiple theories to explain the pathophysiology of tendinopathy; the most common are the mechanical, inflammation, apoptosis, and vascular and neurogenic theories [5,6,7]. Both genetic and environmental factors are involved in tendon disorders pathogenesis and an important interplay between biological and mechanical factors could be detected in tendon disorders physiopathology [5,6,7].

The pathological process seems to be initiated by repetitive tendon overload, resulting in structural injury to the microscopic collagen fibrils. The diagnosis of tendinopathy is based on clinical symptoms and the patient’s history of localized tendon pain and stiffness after activity. In the early stages of tendinopathy, patients may endure discomfort and normally continue to work, play sports, or do daily activities, as they often experience no symptoms once warmed up. In addition, patients often describe a continuum of initial symptoms, which can then evolve into constant, debilitating pain during activities.

Medical treatment aims to reduce and eventually eliminate pain, decrease inflammation, promote tendon healing, and restore joint function with a consequent return to sports activity as soon as possible. In each case, a personalized, patient-centred approach should be considered.

First-line treatment should encompass an individualized tailored loading program, referred to as the precision tendinopathy management plan [8]. This approach may include isotonic, isometric and eccentric energy storage loading versus energy storage and release.

In mild forms, a regimen called RICE, an acronym for “Rest, Ice, Compression and Elevation”, eventually associated with the use of non-steroidal anti-inflammatory drugs (NSAIDs), should be employed to limit the pain and additional damage. The use of Extracorporeal Shock Wave Therapy (ESWT) has been associated with significantly better scores than comparison therapy on the visual analogue pain scale [9]. After the failure of conservative strategies such as anti-inflammatory medications, rest, ice massage, and immobilization, corticosteroid injections are sometimes used to treat tendinopathies, although some research suggests that they could lead to tendon rupture [10]. Moreover, numerous studies have demonstrated the efficacy and safety of polydeoxyribonucleotide (PDRN) in treating tendinopathies by subcutaneous peritendinous infiltration as an alternative treatment to corticosteroids and NSAIDs. PDRN is a proprietary and registered drug with several beneficial effects, including tissue repairing, anti-ischemic action and anti-inflammatory properties. It contains a mixture of deoxyribonucleotides with molecular weights ranging between 50 and 1500 KDa, derived from a controlled purification and sterilization process of sperm DNA from Oncorhynchus mykiss (Salmon Trout) or Oncorhynchus keta (Chum Salmon) [11]. In scientific literature, PDRN has been shown to stimulate the proliferation of a wide range of cell types involved in the healing process of surgical wounds, both in vitro and in vivo [12,13].

The most relevant mechanism of action of PDRN is the activation of adenosine A2A receptors, which play a central role in modulating inflammation, oxygen consumption, ischemia, cell growth and angiogenesis. Indeed, PDRN has been shown to promote the binding of adenosine to A2A receptors, resulting in an increased VEGF expression, cell differentiation, fibroblast maturation and collagen synthesis, thus accelerating granulation tissue formation and the repair process in wound healing [14].

PDRN suppresses the secretion of pro-inflammatory cytokines and exhibits anti-inflammatory effects. Thellung et al. compared PDRN to adenosine in primary cultures of human skin fibroblasts, proving that both induced cell growth.

Simultaneous incubation with 3,7-Dimethyl-1-propargylxanthine (DMPX), an adenosine A2 receptor antagonist, abolished the effects of PDRN, thus hypothesizing that PDRN may act preferentially on the adenosine A2A receptor [13].

Frequently, damaged or hypoxic tissue cannot undergo “de novo” DNA synthesis. PDRN generates nucleotides and nucleosides that can contribute to DNA formation, therefore reactivating normal cell proliferation and growth patterns, as demonstrated by Sini et al. [15].

The stimulating effect of PDRN on cell growth has also been studied in cultured human osteoblasts [16]. In addition, PDRN was also tested in primary chondrocytes [17], where it induced physiological extracellular matrix accumulation with a reduction in the action of matrix metalloproteinases 2 and 9, consequently leading to decreased proteoglycan degradation. The synergistic action of PDRN and glucosamine in reducing extracellular matrix gene expression reduces its degradation [18]. This evidence designates the DNA-derived drug as a potential therapy in the regenerative treatment of cartilage damage.

Furthermore, PDRN has been suggested to accelerate bone tissue repair and growth [16]. The improvement in angiogenesis has been confirmed by an increased expression of CD31, transglutaminase-II and angiopoietin, factors contributing to new vessel formation. PDRN reduces serum levels of Tumor Necrosis Factor (TNF-α) and increases VEGF and nitric oxide production in wounds, displaying a marked systemic effect.

In an experimental model of peripheral artery occlusive disease, Bitto proved the ability of PDRN to promote angiogenesis. PDRN boosted a robust blood flow regeneration with a marked increase in VEGF expression, while DMPX abrogated the drug’s beneficial effects [19].

**Figure 1:**
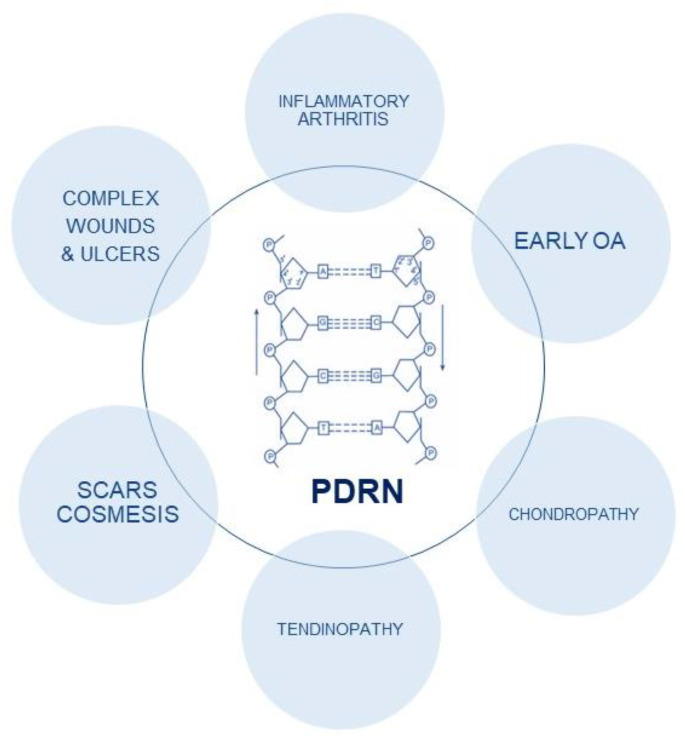
PDRN structure and biological effects.

The activation of the adenosine A2A receptor results in an anti-inflammatory effect, and it represents an attractive target for anti-inflammatory agents. PDRN was evaluated in collagen-induced arthritis: in this experimental paradigm, PDRN was shown to improve clinical signs of arthritis, reduce histological damage and decrease the amount of several pro-inflammatory cytokines both in cartilage and blood, while increasing the expression of anti-inflammatory cytokines such as interleukin-10 (IL-10) (Figure 1). PDRN restored the histological features, blunted inflammatory and apoptotic proteins expression and preserved Bcl-2 expression [20]. The A2A receptor has been indicated as a therapeutic target to modulate ischemic insult.

Studies performed on experimental models of testicular torsion and varicocele [21,22] concluded that PDRN can reduce the inflammatory cascade and rebalance the apoptotic mechanism, consequently improving spermatogenesis and protecting histological damage. The latter could also be attributed to the ability of PDRN to limit ischemic reperfusion injury, by increasing VEGF expression and angiogenesis, as observed in kidneys [23].

Studies investigating the safety of PDRN have confirmed that PDRN does not result in any mortality or have any toxic effect on the liver, lungs, brain, skeletal muscles or heart [24].

This study aims to summarize the current evidence about PRDN’s clinical effectiveness in the management of tendinopathies.

## 2. Methods

The study was conducted using the Preferred Reporting Items for Systematic Reviews and Metanalyses (PRISMA) methods.

### 2.1. Literature Search and Study Eligibility

The first step consisted of a scoping literature search performed on studies published from January 2012 to November 2022 by one reviewer, D.B., using the following databases: OVID-MEDLINE^®^, EMBASE, SCOPUS, Web of Science, Google Scholar and PubMed. We selected an initial pool of potentially relevant studies to investigate the use of PDRN in tendinopathies.

The search strategy included the following terms: (PDRN” [MeSH Terms] OR “polydeoxyribonucleotides” [All Fields]) AND ((“tendinopathies” [MeSH Terms] OR (“tendon diseases” [All Fields])).

The second step consisted of revising the records to identify studies dealing with peritendon or intratendinous injection of PDRN in tendinopathies.

### 2.2. Study Quality Assessment and Risk of Bias of the Included Studies

The quality of each included study was assessed according to the AAOS clinical practice guideline and review methodology version 2 [25]. The following points were evaluated: sample size and features; description of inclusion end exclusion criteria; the number of lost to follow-up; prognostic factors evaluation; outcome evaluation; appropriate statistical analysis; references of the study; data evaluation; the presence of bias; the presence of confounding factors; follow-up length.

Based on the depicted flaws and the study design, the quality of each study included in this systematic review was defined as follows: high quality (<2 flaws); moderate quality (≥2 and <4 flaws); low quality (≥4 and <6 flaws); very low quality (≥6 flaws).

Two authors (B.D. and P.A.) independently evaluated all the studies. In case of disagreement between them, a new combined evaluation was performed. Two senior authors (M.B. and S.G.) finally approved the quality assessment procedure.

Publication bias could not be assessed by a funnel plot, considering the very low number of patients in each study.

## 3. Results

### 3.1. Study Selection

**Figure 2:**
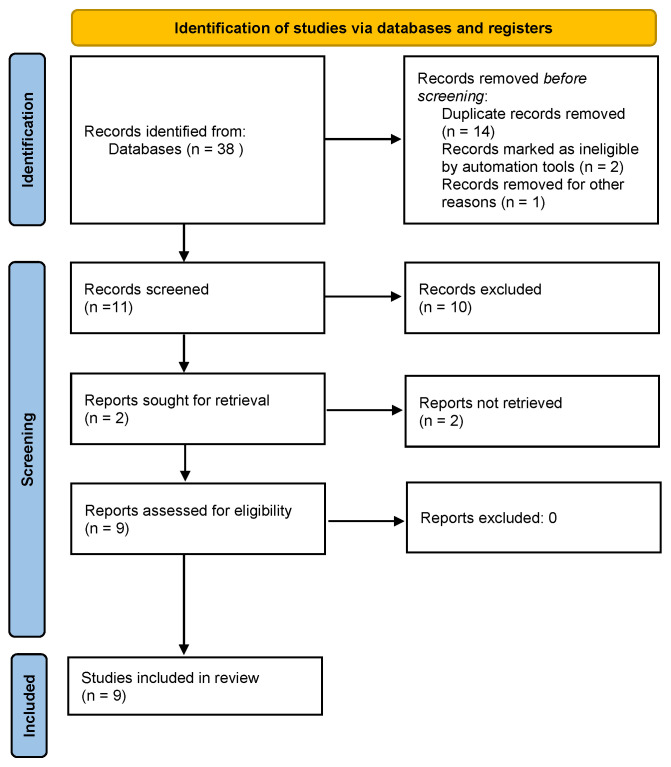
PRISMA flow diagram.

The OVID-MEDLINE^®^, EMBASE, Cochrane Library, SCOPUS, Springer Link, Web of Science, Google Scholar and PubMed database searches provided a total of 38 studies for potential inclusion in the review. After adjusting for duplicates, 21 studies remained. Of these, 12 studies were discarded after reading titles and reviewing abstracts. A total of two in vivo studies and seven clinical studies were finally included in the present review. The PRISMA flow diagram is reported in Figure 2. 

### 3.2. Study Quality and Characteristics

The study characteristics are summarized in Table 1 and Table 2. The process of quality assessment, performed according to the AAOS clinical practice guideline and review methodology version 2, depicted the following results: five studies out of seven (71.43%) were classified as high-quality studies, whereas the reaming two studies out of seven (28.57%) were classified as moderate-quality studies (Table 2).

### 3.3. Effectiveness of PDRN in the Management of Tendinopathies

#### 3.3.1. Achilles Tendinopathy

In a study conducted on rat models, Kang et al. [26] demonstrated the efficacy of PDRN to promote collagen synthesis, secretion of numerous growth factors and restoration of tensile strength in Achilles tendon injury. The Achilles tendon was transected and repaired by the modified Kessler’s method.

Experimental procedures were performed by splitting subjects into two different groups: the control group received intraperitoneal injections of normal saline, while the experimental group was treated with PDRN in the same amount.

In this study, the PDRN-treated group showed an increase in cross-sectional area and significant differences in maximum load and tensile stress values, probably due to intensified collagen synthesis. In addition, tendons in the PDRN group were found to be more resistant to mechanical insults and stress due to high levels of type 1 collagen.

Based on a previous study of the elevation of TGF-b1 mRNA in the injured tendon [35], PDRN could influence the expression of TGF-b1, which plays an important role in the healing process of tendon injury by promoting fibroblasts recruitment and the secretion of ECM proteins. This aspect is closely related to the synthesis of type 1 collagen, which is important for the restoration of tensile strength [36]. Moreover, a higher expression of FGF, VEGF, and TGF b1 was detected in the experimental group, in agreement with reports about their stimulation by the adenosine A2A receptor [14,37].

In a study by Rho, PDRN was shown to be effective as an anti-inflammatory agent and suppressor of apoptotic cell death in several disorders [27]. Forty rats were divided into five groups (eight rats per each group): control group, Achilles tendon-injured rats, Achilles tendon-injured rats with the application of 2 mg/kg PDRN, Achilles tendon-injured rats with the application of 4 mg/kg PDRN and Achilles tendon-injured rats with the application of 8 mg/kg PDRN.

Tendon injury induces an increase in pro-inflammatory agents such as TNF-α and IL-6 as a result of the local inflammatory response. Delayed treatment of Achilles tendon injury leads to degenerative changes in the tissue due to excessive apoptotic activity [27]. Caspases are fundamental in the process of programmed cell death. Caspase-3 can be considered an effector caspase since it breaks down the proteins in the cell; on the other hand, caspase-8 and caspase-9 act as initiators of the apoptotic process [38]. The cleaved form of caspases activates the apoptosis pathway, causing DNA degradation, which in turn leads to cell death [38]. Bcl-2 and Bcl-2-associated X (Bax) are two proteins belonging to the B-cell lymphoma 2 (Bcl-2) family. Bcl-2 suppresses the apoptotic process and the expression of pro-apoptotic proteins, while Bax induces apoptosis. When pro-apoptotic proteins are more expressed than antiapoptotic ones, the apoptotic process begins in the tendon, eventually promoting its damage.

In this study, an analysis of histological alterations was conducted using hematoxylin and eosin staining. Immunohistochemistry was performed for cleaved caspase-3 and cleaved caspase-9 positive cells. Enzyme immunoassay was used to detect TNF-α, IL-6 and cAMP concentrations. Western blot was employed for the detection of proteins such as cAMP response element-binding protein (CREB), protein kinase A (PKA), Bax and Bcl-2.

In the current results, after Achilles tendon injury, the percentages of cleaved caspase-3- and caspase-9-positive cells increased, as well as the Bax-to-Bcl-2 ratio, thus suggesting that Achilles tendon damage exacerbated apoptosis. In contrast, PDRN application decreased the rates of cleaved caspase-3- and caspase-9-positive cells and the Bax-to-Bcl-2 ratio in Achilles tendon-injured rats.

Von Frey filament and plantar tests were used to determine the pain threshold. In the current results, the tactile threshold of the von Frey filament test and paw withdrawal latency of the plantar test were reduced after the Achilles tendon injury. These results indicate that mechanical allodynia and thermal hyperalgesia occurred after Achilles tendon injury. In contrast, treatment with 8-mg/kg PDRN alleviated mechanical allodynia and relieved thermal hyperalgesia after Achilles tendon insult.

PDRN treatment reduced the expression of proinflammatory cytokines such as IL-1β, IL-6 and TNF-α. In the present results, TNF-α and IL-6 concentrations increased after Achilles tendon injury, indicating that the Achilles tendon injury was exacerbated due to the overproduction of TNF-α and IL-6. However, TNF-α and IL-6 concentrations decreased after PDRN application in rats with an Achilles tendon injury.

The present study showed that PDRN treatment reduced pain sensitivity and improved histological degeneration in rats with an Achilles tendon injury. Moreover, PDRN treatment suppressed inflammation and apoptosis in rats with an Achilles tendon injury.

In a case report described by Lim [28], a female patient with posterior tibial tendon dysfunction (PTTD), who underwent syndesmotic surgery, received prolotherapy with PDRN. Ankle pain persisted after screw removal, and oedema for about 1 month was due to long-term NSAID administration. PTTD is an acquired deformity that can cause flatfoot and pain. The primary function of the PTT is to stabilize the hindfoot against eversion and valgus forces. It assists the Achilles tendon in plantar flexion and acts as the main invertor of the foot. After PDRN administration in PTT, NRS scores decreased from 8 to 5 at the 1-week follow-up. PDRN injection was repeated four times at 1-week intervals. The patient reported a significant reduction in pain, with a decrease in NRS scores from 5 to 1.

Through VEGF stimulation and fibroblast maturation and differentiation, PDRN accelerates the repair process. Additionally, PDRN lowers the expression of the pro-inflammatory cytokines IL-6 and TNF-alpha [28], a potential mechanism for its rapid anti-inflammatory effect in PTT. In conclusion, PDRN prolotherapy represents an effective and safe treatment option for PTTD.

#### 3.3.2. Plantar Fasciitis

In a prospective randomized clinical trial conducted by Kim [29], the safety and efficacy of PDRN injection were demonstrated for patients with chronic plantar fasciitis. Forty patients were randomly assigned to PDRN injection or normal saline injection. Injections were performed weekly for three weeks. The visual analogue scale (VAS) for foot pain and the Manchester-Oxford Foot Questionnaire (MOXFQ) were used to assess pain just before initial treatment when the two groups had no significant differences, and at 4 weeks and 12 weeks after treatment. The VAS and MOXFQ scores of the PDRN group were better than those of the placebo group at 4 and 12 weeks after treatment. No injection-related complications were noted in this study.

In this case, the stimulatory effect of VEGF in inducing angiogenesis in the healing process should be considered, as well as the increase in the anti-inflammatory cytokine IL-10 and the decrease in the pro-inflammatory cytokines TNF-α and IL-6.

Along the same line, it is possible to mention a comparative study by Lee [30], in which 44 patients with plantar fasciitis were randomly assigned to PDRN or corticosteroid injection. The study showed no significant differences after 6 months of follow-up using the same pain scores.

#### 3.3.3. Rotator Cuff Tendinopathy

PDRN prolotherapy can improve the conservative treatment of painful rotator cuff tendinopathy for patients aged 30 to 75 years with rotator cuff lesions in the form of tendinosis, partial tear (involvement < 50% of the tendon) on ultrasonography (US) or magnetic resonance imaging (MRI), with symptoms that persisted for at least 3 months, refractory to other conservative methods such as physical therapy and exercise therapy. In a retrospective study, Ryu et al. [31] selected 32 patients who were treated with PDRN.

The outcome was assessed with the use of the VAS score, shoulder pain and disability index (SPADI), single assessment numeric evaluation (SANE) and the possible adverse effects. One week after treatment, the VAS score was lower than the one measured before treatment and remained stable at 1 month and 3 months after treatment. Thus, the pain score did not change from 1 week to 3 months after treatment. Shoulder function evaluated by SANE over one week after treatment improved the most at 1 month and 3 months after treatment. In the same way, disability assessed by SPADI after 1 month and 3 months was lower than the one measured at 1 week. No complications of any kind occurred during the treatment procedure.

#### 3.3.4. Epicondylitis

Shim et al. compared the effectiveness of different conservative treatments in a randomized controlled trial [32]. In total, 69 patients with chronic LE were enrolled in this study and divided into 3 groups (6 patients were excluded and each group included 21 patients). All patients in the three groups were taught to perform extensor muscle strengthening exercises with counterforce braces (EX). Group 2 was treated with PDRN, while group 3 received extracorporeal shock-wave therapy (ESWT). The VAS pain score, Mayo elbow performance score (MEPS) and ultrasonographic examinations were checked before, 6 weeks after and 12 weeks after the treatments. The results showed that the mean VAS and MEPS of LE patients improved after the use of EX alone or combined with PDRN injections and ESWT. Ultrasound findings also improved after the treatments. Among the three groups, the mean MEPS of group 2 improved significantly more than groups 1 and 3 at 6 weeks, and more than group 1 at 12 weeks. The mean common extensor tendon depth (CETD) on ultrasonography in group 2 increased significantly more than groups 1 and 3 at 6 weeks, and that of groups 2 and 3 increased more than group 1 at 12 weeks. We can say that PDRN injections combined with EX showed greater improvement in mean MEPS and mean CETD compared to either EX alone or EX combined with ESWT for LE in the 12-week follow-up.

In the cases reported by Lee [33], patients with lateral epicondylitis (LE) received ultrasound-guided PDRN injections into the common extensor tendon and showed improvement in symptoms. Two weeks after the PDRN injection significant pain relief was reported; additionally, no significant changes in tendinosis resulted in a US follow-up. This effect is thought to be attributable to the anti-inflammatory action of PDRN, which reduces pro-inflammatory cytokines such as IL-1 and 6, and increases the levels of anti-inflammatory cytokines such as IL-10, as demonstrated in previous studies.

#### 3.3.5. Pes Anserine Tendinopathy

In a case report described by Mun et al. [34], a female patient with Pes anserine (PA) bursitis successfully received a PA bursa PDRN injection for left medial knee pain. The patient reported chronic refractory pain in the inner knee area during aggravating daily activities.

At the time of admission, the patient was unable to walk because of severe pain, rated 7/10 on the NRS. No improvements occurred after physical therapy (bandages) and NSAIDs. The patient refused to use glucocorticoid bursa injection after the effects and side effects. At the 1-week follow-up after the PA bursa PDRN injection, the NRS score had decreased from 7 to 2. At the 2-week follow-up, the patient reported significant pain reduction with decreased NRS scores from 2 to 0. Follow-up was continued for more than eight months, and the patient recovered completely. She had no pain and had a full range of motion in the left knee during walking. No adverse reactions or side effects were observed.

## 4. Discussion

PDRN have shown a good safety profile and revealed effectiveness in the treatment of tendon disorders. The clinical effectiveness of this therapeutic drug relies on a solid scientific rationale, as depicted in preclinical studies.

In in vivo studies, PDRN (8 mg/kg) showed no toxic effect in the brain, liver, lungs skeletal muscle and heart and did not cause mortality.

In clinical trials, PDRN safety and tolerability were excellent. Moreover, a post-marketing surveillance study involving selling more than 300,000 PDRN-dispensed prescriptions confirmed the excellent safety profile of the drug [34].

The progressive spread in clinical practice of PDRN might reduce the use of corticosteroids in the treatment of tendon disorders, thus also improving the treatment of tendinopathies in diabetic patients, who could not receive local corticosteroid injections because of their underlying pathology.

The main limitations of the current literature are the lack of combined clinical protocols. Although in vivo studies [39,40] have shown a synergic effect of PDRN plus extracorporeal shockwave therapy (ESWT) [39] and PDRN plus microcurrent [40] in the treatment of rotator cuff tendon tear in a rabbit model. Based on these findings, future multicenter randomized clinical studies, with a bigger sample size and promoting integrated therapeutic protocols, are needed.

The integrated protocols, including PDRN, ESWT and biophysical stimulation with pulsed electromagnetic fields (PEMFs) [41,42,43,44,45,46,47], are highly encouraged considering the clinical effectiveness of such integrated approaches.

It is important to note, furthermore, that PDRN and PEMFs act on the same molecular target, i.e., A2A receptors; therefore, a synergism between these two therapies might improve the therapeutic effect in tendon disorders [41,42,43,44,45,46,47].

## 5. Conclusions

PDRN are a valid emerging therapeutic drug in the treatment of tendinopathies. Further multicentric randomized clinical studies are needed to better define the therapeutic role of PDRN, especially in combined clinical protocols including PDRN, ESWT and biophysical stimulation with pulsed electromagnetic fields (PEMFs) [41,42,43,44].

## Figures and Tables

**Table 1:** In vivo studies.

Study	Design	Treated Tendinopathy	Number of Patients	PDRN Protocol	Main Findings
Kang et al.—2018 [26]	In vivo randomized controlled study	Achilles tendon injury	36 male rats	The rats received daily intraperitoneal administration of polydeoxyribonucleotide (8 mg/kg/day for 1, 2, or 4 weeks)	↑ Resistant to mechanical stress ↑ Stored energy
Rho et al.—2020 [27]	In vivo randomized controlled study	Achilles tendon injury	40 rats		↑ The tactile threshold for the von Frey filament test tactile withdrawal latency ↓ The concentration of IL-6 and ↓ TNF-α ↑ Expression of cAMP ↑ Phosphorylation of CREB and PKA ↓ Expression ratio of Bax/Bcl-2

↑ = upregulation; ↓ = downregulation.

**Table 2:** Main findings of the included clinical studies.

Study	Design and Quality Assessment	Treated Tendinopathy	Number of Patients	PDRN Protocol	Main Findings
Lim et al.—2016 [28]	Case report (moderate quality)	Posterior Tibial Tendon Dysfunction (PTTD)	1 Female patient (age: 67)	PDRN injection repeated 4 times at 1-week intervals	↓ NRS score and pain, swelling and tenderness, complications
Kim et al.—2015 [29]	Prospective randomized trial (High quality)	Chronic plantar fasciitis	20 patients (male: 7)	PDRN injection performed weekly for three weeks	↓ VAS and MOXFQ scores No complications
Lee et al.—2020 [30]	Prospective randomized trial (High quality)	Plantar fasciitis	44 male patients	PDRN injection performed weekly for three weeks	↓ VAS and MOXFQ scores No complications
Ryu et al.—2018 [31]	Retrospective Study (High quality)	Chronic rotator cuff disease	32 patients (17 males)	PDRN injection performed weekly for four weeks	↓ VAS score, pain, SPADI ↑ Function and SANE No complications
Shim et al.—2021 [32]	Randomized controlled trial (High quality)	Lateral epicondylitis	69 patients (Male: 33; Mean age: 51.07)	Group 1: brace only Group 2: brace +PDRN injection Group 3: brace + ESWT	PDRN+ Brace: ↓ VAS score, ↑ MEPS, ↑ ultrasonographic findings, compared to control groups
Lee et al.—2018 [33]	Case series (High quality)	Lateral epicondylitis	2 patients (Male: 2; mean age: 62)	US-guided PDRN injections were made into the common extensor tendons	↓ Pain with decreased NRS ↓ Hypervascularity of common extensor tendon Improvement in the LE symptoms without any complications
Mun et al.—2017 [34]	Case report (Moderate quality)	Pes anserine bursitis	1 female patient (Age: 50)	US-guided PDRN injections were made into PA bursa	↓ Pain with decreased NRS ↑ ROM

↑= upregulation; ↓= downregulation.

## Data Availability

Not applicable.
